# Effect of Family Medical History of Hypertension and Its Interaction with Diabetes on an Additive Scale on the Diagnosis of Hypertension Among Middle-Aged and Older Adults in India: Longitudinal Ageing Study in India

**DOI:** 10.3390/healthcare14203538

**Published:** 2026-10-20

**Authors:** Ghazi I. Al Jowf, Waquar Ahmed

**Affiliations:** Department of Public Health, College of Applied Medical Sciences, King Faisal University, Al Hofuf 37912, Saudi Arabia

**Keywords:** additive interaction, synergistic effect, family medical history, hypertension, diabetes, middle-aged and older adults, longitudinal aging study in India

## Abstract

**Introduction:** Among individuals diagnosed with type 2 diabetes, the family medical history remains an independent risk factor for elevated blood pressure. In fact, the individual effect of diabetes on the risk of hypertension could not truly reflect the actual effect of exposures, and there might be an interaction with a family history of hypertension. The coexistence of these two factors may synergistically increase the risk of hypertension, leading to adverse health outcomes. This study aimed to examine the effect of the additive interaction between a family medical history of hypertension and diabetes on the diagnosis of hypertension among individuals aged 45 years and older in India. **Methods:** The study used data from the Longitudinal Ageing Study in India (LASI) Wave 1 (2017–2018), comprising a total sample size of 58,667 individuals aged 45 years and above. An additive model was employed to estimate the interaction effect of the family medical history of hypertension with diabetes on the diagnosis of hypertension by calculating three measures of additive interaction: the relative excess risk due to interaction (RERI), attribution proportion (AP), and synergy index (S). **Results:** Individuals with a history of both the parents and siblings had an AOR (adjusted odds ratio) of 2.1 and 3.3 for hypertension. In the adjusted model, the RERI, AP, and S for hypertension were 3.09 (95% CI: 1.28–4.89), 37.0% (0.37; 95% CI: 0.22–0.52), and 1.73 (95% CI: 1.31–2.27), respectively, which indicates that there is a significant positive interaction effect between a family history of hypertension and diabetes on the diagnosis of hypertension. **Conclusions:** The study findings strongly indicate that a family medical history of hypertension and diabetes has a significant and positive interaction effect on the risk of hypertension. Therefore, it is imperative to consider both the family history and diabetes in hypertension risk assessments and develop targeted primary and secondary prevention strategies for specific vulnerable subpopulations.

## 1. Introduction

High blood pressure is the leading heritable risk factor for cardiovascular disease [1]. Twin and family studies have estimated the heritability of hypertension, ranging from 30% to 60% [2,3,4]. The genetic inheritance of hypertension is predominantly multifactorial, influenced by environmental factors such as high salt intake and a lack of physical activity [5,6]. A recent study indicated that family history is an influential factor for the risk of developing hypertension [2]. Specifically, a family history of hypertension serves both as a predictor and a risk factor for hypertension [7]. Additionally, prior research has confirmed the familial aggregation of blood pressure [8]. Previous studies have shown that the prevalence of hypertension increases with the number of affected family members, and individuals with hypertensive first-degree relatives are at a higher risk of developing hypertension compared to those with hypertensive second-degree relatives [9,10]. Moreover, the consistent association of blood pressure between mothers and their offspring suggests that mothers may play a crucial role in the primary prevention of hypertension [8].

Genome-wide association studies (GWASs) on blood pressure variability [11] and longitudinal blood pressure traits [12] have identified novel loci associated with these traits [11,12]. In a recent study, within a multiancestry cohort comprising more than 21,000 US adults, the genome-wide systolic blood pressure (SBP) polygenic risk score (PRS), computed using the 1.08 million variants identified from SBP genome-wide association studies (GWASs), has shown a significant association with blood pressure (BP) traits and adverse cardiovascular events [13]. Notably, a one-standard-deviation (SD) increase in the SBP PRS was found to be significantly associated with elevated SBP and an increased risk of hypertension [13]. Moreover, the SBP PRS showed an association with a seven percent increase in the 10-year risk of incident cardiovascular events after adjusting for traditional cardiovascular risk factors [13].

In a prior study, individuals with diabetes were observed to have a notably elevated incidence of hypertension [14]. According to a prior study based on National Health and Nutrition Examination Survey (NHANES) data, it was found that during the period of 2011–14, 36.6% of adults with prediabetes had hypertension. Furthermore, between 1988–94 and 2011–14, there was a notable rise in the prevalence of hypertension among adults with prediabetes, with an increase of 9.7 percentage points [15]. Additionally, a previous study demonstrated that diabetes is significantly associated with an elevated risk of cardiovascular disease (CVD), and risk is further increased with the coexistence of hypertension [16].

High blood pressure is a leading risk factor for coronary heart disease, heart failure, and stroke [17,18,19]. Notably, a recent study utilizing Mendelian randomization analysis revealed positive causal associations for higher blood pressure in 14 cardiovascular conditions, including ischemic heart disease (IHD) and stroke [20]. Additionally, the study demonstrated that the genetically predicted blood pressure was found to be positively associated with total cardiovascular diseases (CVD) [20].

High blood pressure is a heritable trait [21]. Moreover, a prior study has shown that in individuals diagnosed with type 2 diabetes, family history remains an independent risk factor for elevated blood pressure [22]. Additionally, in a previous study, a family history of hypertension and diabetes or impaired glucose tolerance were found to be independently associated with an increase in mean blood pressure [23]. The positive family history of hypertension among individuals with diabetes is likely to be an underestimate, particularly for hypertension, which may often remain subclinical with damage to target organs. In fact, the individual effect of diabetes on the risk of hypertension could not truly reflect the actual effect of exposures, and there might be an interaction with the family history of hypertension, which may lead to synergistic effect on the risk of hypertension. This study examines the interaction on an additive scale to determine whether the combined effect of a family medical history of hypertension and diabetes on the risk of hypertension is greater or smaller than the sum of their individual effects. Assessing the interaction on an additive scale has relevance to public health because it would identify subgroups in which targeted preventive interventions could potentially have greater impact. With the prevalence of hypertension and diabetes continuing to increase, particularly in low- and middle-income countries (LMICs) where healthcare resources are often constrained, understanding the broader implications of their coexistence is increasingly important. However, there exist a dearth of literature concerning the interaction effect of a family medical history of hypertension with diabetes on the risk of hypertension in LMIC settings, particularly in India [24]. Using nationally representative data, the present study addresses this concern and provides population-level evidence beyond that obtained from conventional health-facility-based studies. The study is particularly relevant in the context of India’s rapidly aging population, as it may contribute to a better understanding of the combined effect of familial predisposition and diabetes on hypertension risk. Furthermore, these findings may provide the evidenced-based information to guide the development of targeted prevention and intervention strategies and support efforts to address the healthcare challenges associated with the coexistence of diabetes and a family history of hypertension among middle-aged and older adults. Therefore, the current study aimed to study the additive interaction between a family medical history and diabetes on the risk of hypertension. Additionally, we assessed the association of different categories of the family medical history with hypertension. It is imperative to consider both family history and diabetes in hypertension risk assessments and develop targeted primary and secondary preventive strategies for specific vulnerable subpopulations.

## 2. Methods

### Data

The data from the Longitudinal Ageing Study in India (LASI) Wave 1 (2017–2018) were used in this study. The survey collected data on the health, economic, and social factors and consequences of India’s population aging. The LASI is a full-scale, nationally representative survey that included 72,250 individuals aged 45 years and older and their spouses (irrespective of age) across all states and union territories (UTs) of India except Sikkim. The LASI uses a multistage stratified area probability cluster sampling to select the eventual units of observation. This study presents scientific evidence on chronic health conditions, biomarkers, symptom-based health conditions, and functional and mental health. The LASI survey was conducted with a three-stage sampling design in rural areas and a four-stage sampling design in urban areas. In each state/UT, in the first stage, Primary Sampling Units (PSUs) were selected, and in the second stage, villages in rural areas and wards in urban areas were selected in the selected PSUs. In the third stage, households were selected from each selected village; however, sampling in urban areas involved an additional stage, i.e., the random selection of one Census Enumeration Block (CEB) in each urban area. In the fourth stage, households were selected from each CEB. The main goal was to select a representative sample at each stage of sample selection. The detailed methodology and extensive information on the survey’s design and data collection are available in the report. The present study is based on 65,562 respondents aged 45 years and above. After removing all the cases (6895) with missing information in any of the selected variables, the total sample size for the analysis was 58,667 respondents.

## 3. Measures

### 3.1. Outcome Variables

The main outcome variable was self-reported hypertension. In the study, respondents were asked, “Has any health professional ever diagnosed you with high blood pressure or hypertension?”. The responses were coded as ‘no’ and ‘yes’. In LASI, respondents were asked additional questions to those who reported being diagnosed with a disease by a medical professional, including the diagnosing physician, the date of diagnosis, and whether they are currently receiving treatment. Participants were asked, “In order to treat or control your blood pressure or hypertension, are you currently taking any medications?”. Interaction effects were estimated in two different study samples, (1) self-reported hypertension and (2) individuals with hypertension who were taking treatment.

### 3.2. Key Explanatory Variable

The main explanatory variable was self-reported diabetes and a family medical history of hypertension. In the study, respondents were asked, “Has any health professional ever diagnosed you with diabetes?”. The responses were coded as ‘no’ and ‘yes’. In LASI, respondents were asked an additional question concerning medication, to those who reported being diagnosed with a disease by a medical professional including whether they are currently receiving treatment. Participants were asked, “In order to control your diabetes or high blood sugar, are you currently taking any medication?”.

In the LASI, to understand the genetic risk factors for hypertension, information was collected about the respondent’s family medical history; family medical history of the father, mother, brother, and sister were selected for the analysis.

### 3.3. Other Covariates

Age was coded as 45–54 years, 55–64 years, 65–74 years, and 75+ years. Gender was coded as male and female. Education was recoded as no education, primary, secondary, and higher. Marital status was coded as currently married, widowed, others, and not in a union. Working status was coded as never worked, currently working, and currently not working. Alcohol use was coded as ‘no’ and ‘yes’; smoking and chewing tobacco were coded as ‘never’, ‘former’, and ‘current’. The body mass index (BMI) was computed by dividing the weight (in kilograms) by the square of the height (in meters). BMI was coded according to the criteria of the World Health Organisation’s classification, as underweight (<18.5 kg/m^2^), normal weight (18.5–24.9 kg/m^2^), overweight/pre-obesity (25.0–29.9 kg/m^2^), and obesity (≥30.0 kg/m^2^), and for the analysis, overweight and obesity were combined [25]. The monthly per capita expenditure quintile (MPCE) or consumption quintile was categorized into five quintiles, poorest, poor, middle, rich, and richest. Religion was categorized as Hindu, Muslim, Christian, and Others. The social group (caste) was categorized as Scheduled Castes (SC), Scheduled Tribes (ST), Other Backward Classes (OBC), and others. The place of residence was coded as urban and rural. The regions were categorized as North, Central, East, Northeast, West, and South. In the study, respondents were asked, “Has any health professional ever diagnosed you with high cholesterol”. The responses were coded as no and yes. Respondents were asked, “Has any health professional ever diagnosed you with arthritis?”. The responses were coded as no and yes. Respondents were asked, “Has any health professional ever diagnosed you with stroke?”. The responses were coded as no and yes. Activities of daily living (ADLs) and instrumental activities of daily living (IADLs) were categorized as no and yes [26]. Physical inactivity was coded as physically active and physically inactive [27,28,29].

### 3.4. Statistical Analysis

Multivariable logistic regression was employed to estimate the unadjusted and adjusted estimates, aiming to assess the joint effect of a family medical history of hypertension with diabetes on the diagnosis of hypertension. Moreover, an additive model was employed to assess the additive interaction effect of a family medical history of hypertension with diabetes on the diagnosis of hypertension by estimating three different measures of the additive interaction: the relative excess risk ratio (RERI), attribution proportion due to interaction (AP), and synergy index (S). Interaction on an additive scale means that the combined effect of two exposures is larger (or smaller) than the sum of the individual effects of the two exposures [30,31].

The interaction measures on the additive scale are defined as follows: RERI (excess risk of the outcome due to the interaction between two factors) = OR11 − OR10 − OR01 + 1; AP (proportion of the combined effect that is attributable to the interaction between two factors) = RERI/OR11; S (ratio between combined effect and individual effects) = (OR11 − 1)/(OR10 − 1) + (OR01 − 1). RERI = 0 indicates no interaction, RERI > 0 suggests a positive interaction, RERI < 0 suggests a negative interaction, AP = 0 suggests no interaction, AP > 0 suggests a positive interaction, AP < 0 suggests a negative interaction, S = 1 suggests no interaction, S > 1 suggests a positive interaction, and S < 1 indicates a negative interaction [27,30,31,32].

Bivariate analysis was performed to investigate the prevalence of hypertension and proportion of individuals who were using medication in relation to selected variables. A chi-square test and bivariate analysis were also employed to investigate the prevalence of hypertension concerning the combined effect of a family medical history of hypertension with diabetes. Moreover, multivariable binary logistic regression analysis [33] was employed to establish the association between hypertension and main explanatory variables including diabetes and a family history of hypertension (comprising overall family history, parental (both and any), sibling (both and any), and specific histories related to the father, mother, brother, and sister).

In the current study, the multivariable logistic regression and additive interaction models were adjusted for potential confounding factors, including age, sex, education, working status, marital status, residence, MPCE, religion, caste, region, physical inactivity, smoking, chewing tobacco, alcohol consumption, BMI, ADL, IADL, arthritis, stroke, and high cholesterol. Multicollinearity was assessed using variance inflation factors (VIFs). No substantial multicollinearity was identified. The survey weights were applied during the analysis to account for sample clustering and present population estimates. All the analyses were conducted using Stata version 14.1.

## 4. Results

Table 1 presents the sample characteristics and percentage distribution of hypertension and its treatment among individuals aged 45 years and above. A proportion of around 35% of the participants was 65 years and above. Approximately 54% of the sample population was female. About 51% of the sample had no education during the survey. A large proportion of the sample (73.9%) was in a marital union during the survey. Further, nearly 70% of the participants were living in rural areas.

Table 1 also depicts the prevalence of hypertension among adults aged 45 years and above. The overall prevalence of hypertension was 27.0% (95% CI: 26.2, 27.8), and 71.83% of the individuals with hypertension were taking treatment. Moreover, hypertension was more prevalent among individuals with self-reported diabetes (62.2% vs. 22.2%) and individuals with diabetes who were taking treatment (62.8% vs. 23.1%) than their counterparts. The prevalence of hypertension was higher among individuals aged 65 years and above (34.2% vs. 19.6%), with a secondary education (31.9% vs. 23.9%), and with a higher education (31.2% vs. 23.9%) than their counterparts. Additionally, hypertension was more prevalent among individuals living in urban areas (37.1% vs. 22.7%) than those in rural areas.

The results show that the prevalence of hypertension was higher among physically inactive respondents (30.9% vs. 25.0%) and overweight/obese participants (41.5% vs. 24.2%) than their counterparts. Additionally, the prevalence of hypertension was highly prevalent among individuals with high cholesterol (66.3% vs. 26.1), arthritis (40.8% vs. 25.6), and stroke (61.2% vs. 26.4) than their counterparts. The study findings illustrate that the prevalence of hypertension was higher among individuals with a family history of hypertension (39.4% vs. 23.8%) than those without a family history.

Table 2 presents the prevalence of hypertension among adults aged 45 years and above according to their family medical history.

The prevalence of hypertension was higher among individuals with a medical history of both parents (47.2% vs. 26.5%) and any parent (38.1% vs. 24.9%), respectively, compared with those without a medical history of both parents and any parent. Similarly, the prevalence of hypertension was higher among individuals with a medical history of both siblings (69.7% vs. 26.5%) and any sibling (48.5% vs. 25.3%), respectively, compared with those without a medical history of both siblings and any sibling. Additionally, the prevalence of hypertension was higher among individuals with a medical history of father (38.9% vs. 25.9%), mother (39.5% vs. 25.6%), brother (51.5% vs. 25.6%), and sister (51.4% vs. 26.2%) compared with those without a medical history of father, mother, brother, and sister, respectively.

**Figure 1:**
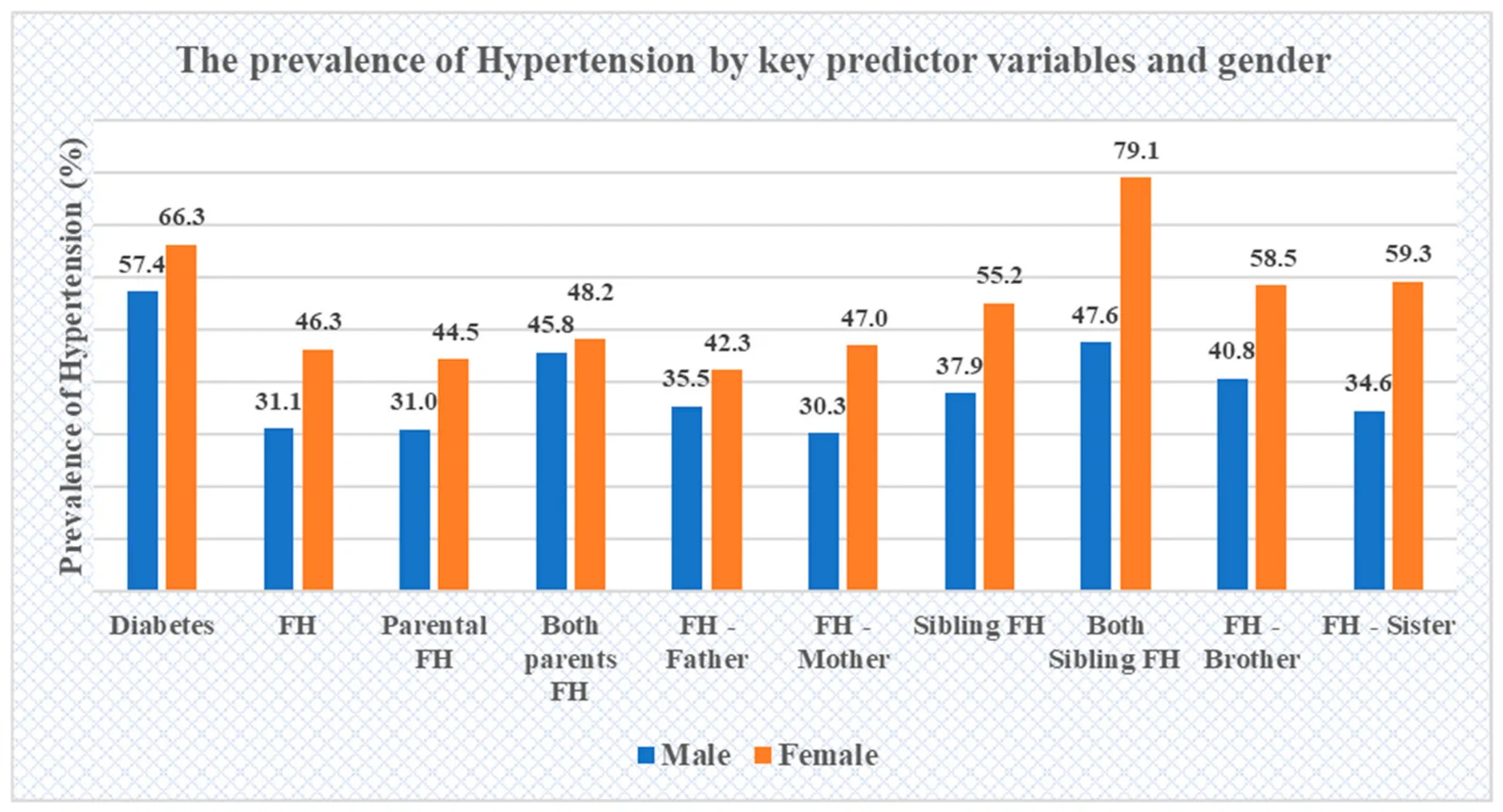
The prevalence of hypertension by key predictor variables and gender. FH, family medical history of hypertension.

**Figure 2:**
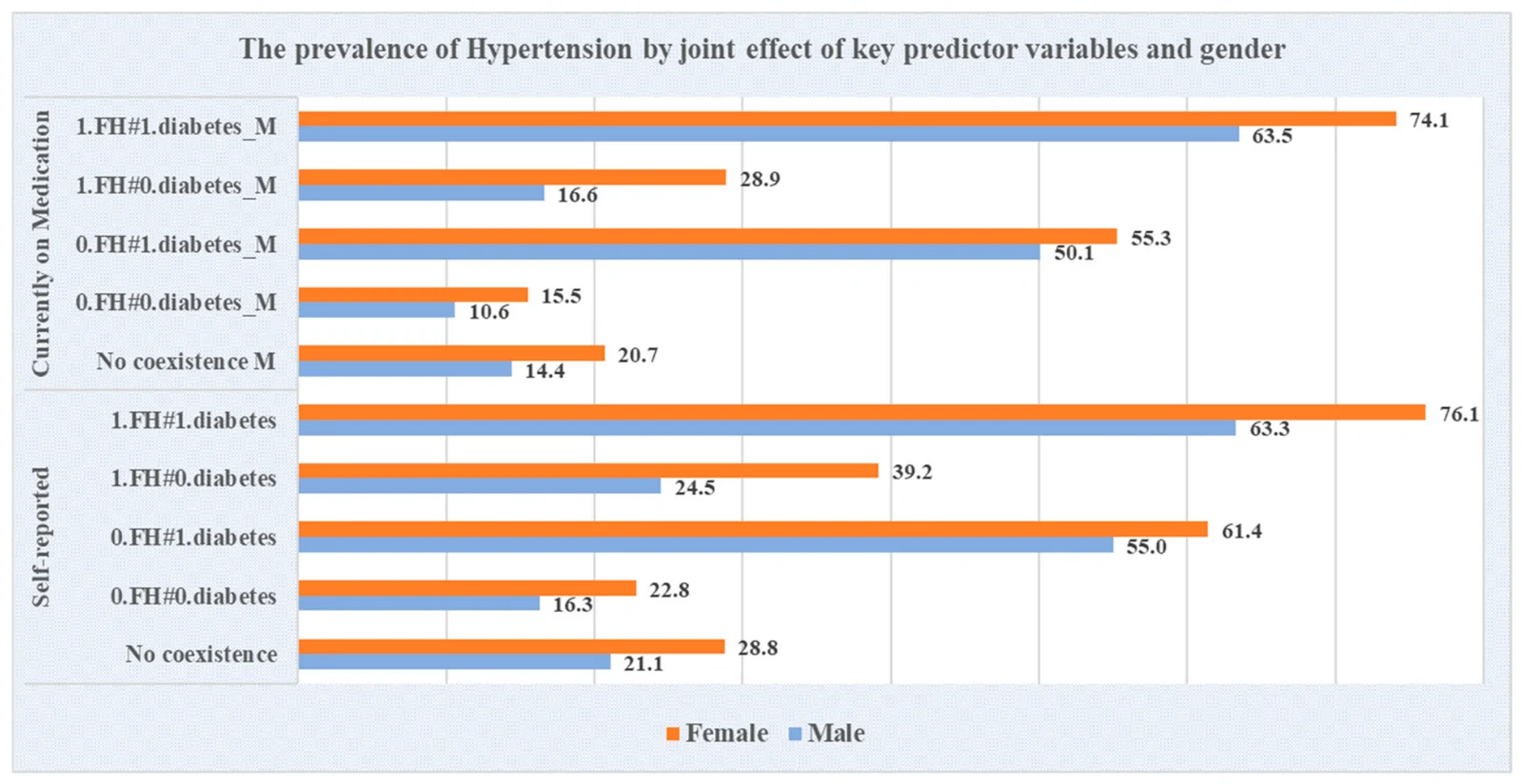
The prevalence of hypertension by joint effect of key predictor variables and gender. diabetes_M, individuals with diabetes who were taking medication using the sample of hypertensive individuals on medication; No-coexistence, either diabetes or family history of hypertension; FH, family medical history of hypertension. 1.FH#1.diabetes_M, presence of family medical history of hypertension # presence of diabetes; 1.FH#0.diabetes_M, presence of family medical history of hypertension # absence of diabetes; 0.FH#1.diabetes_M, absence of family medical history of hypertension # presence of diabetes; 0.FH#0.diabetes_M, absence of family medical history of hypertension # absence of diabetes.

Figure 1 indicates that the prevalence of hypertension was higher among female respondents with a family medical history of hypertension, including parental, sibling, father, mother, brother, and sister medical history of hypertension than among males. The prevalence of hypertension was higher among female respondents with diabetes than males. Similarly, Figure 2 presents that the prevalence of hypertension among the individuals with the coexistence of a family medical history of hypertension and diabetes was higher among female respondents compared to male respondents.

Table 3 represents the prevalence of diabetes by key predictor variables among individuals aged 45 and above.

### 4.1. The Prevalence of Hypertension Based on the Presence or Absence of Family Medical History of Hypertension and Diabetes

#### 4.1.1. Self-Reported Diabetes and Family Medical History of Hypertension

The result presents that the prevalence of hypertension was significantly higher among individuals with diabetes and a family history of hypertension (70.7%, 95% CI: 64.6, 76.1) than those without the coexistence of a family history of hypertension and diabetes (25.3%, 95% CI: 24.6, 26.0), individuals with diabetes and without a family history of hypertension (58.3%, 95% CI: 55.6, 61.0), individuals with a family history of hypertension and without diabetes (32.4%, 95% CI: 30.3, 34.7), and individuals without the presence of both a family history of hypertension and diabetes (19.8%, 95% CI: 19.2, 20.5).

#### 4.1.2. Self-Reported Diabetes (Taking Medication) and Family Medical History of Hypertension

The result presents that the prevalence of hypertension was significantly higher among individuals with diabetes who were taking medication and had a family history of hypertension (64.3%, 95% CI: 57.3, 70.7) than those without the coexistence of a family history of hypertension and diabetes (17.6%, 95% CI: 17.0, 18.3), individuals with diabetes and without a family history of hypertension (48.0%, 95% CI: 45.1, 50.8), individuals with a family history of hypertension and without diabetes (22.9%, 95% CI: 21.1, 24.8), and individuals without the presence of both a family history of hypertension and diabetes (12.9%, 95% CI: 12.4, 13.5).

### 4.2. The Prevalence of Hypertension (Taking Medication) Based on the Presence or Absence of Family Medical History of Hypertension and Diabetes

#### 4.2.1. Self-Reported Diabetes and Family Medical History of Hypertension

The result presents that the prevalence of hypertension was significantly higher among individuals with diabetes and a family history of hypertension (72.7%, 95% CI: 66.0, 78.5) than those without the coexistence of a family history of hypertension and diabetes (25.5%, 95% CI: 24.8, 26.2), individuals with diabetes and without a family history of hypertension (58.0%, 95% CI: 54.8, 61.1), individuals with a family history of hypertension and without diabetes (33.3%, 95% CI: 31.1, 35.5), and individuals without the presence of both a family history of hypertension and diabetes (20.7%, 95% CI: 20.0, 21.4).

#### 4.2.2. Self-Reported Diabetes (Taking Medication) and Family Medical History of Hypertension

The result presents that the prevalence of hypertension (those who were also taking medication) was significantly higher among individuals with diabetes who were taking medication for diabetes and a family history of hypertension (69.7%, 95% CI: 62.6, 76.1) than those without the coexistence of a family history of hypertension and diabetes (17.7%, 95% CI: 17.1, 18.3), individuals with diabetes and without a family history of hypertension (52.8%, 95% CI: 49.6, 56.1), individuals with a family history of hypertension and without diabetes (23.2%, 95% CI: 21.5, 25.0), and individuals without the presence of both a family history of hypertension and diabetes (13.2%, 95% CI: 12.7, 13.8).

Table 4 presents the unadjusted and adjusted logistic regression estimates for hypertension by family medical history of hypertension (overall, parental history, father, mother, sibling history, brother, and sister) and diabetes among individuals aged 45 years and above. In the adjusted model, the results indicate that individuals with diabetes (AOR: 3.9, CI: 3.47–4.38) had 3.90 times higher odds of having hypertension than those without diabetes.

The study findings show that individuals with family history of hypertension (AOR: 1.72, CI: 1.57 1.90) had a significant 1.7 times higher odds of having hypertension than those without a family history. Moreover, individuals with a medical history of any parent and sibling had 1.6- and 2.0-times higher odds of having hypertension, respectively, than those without medical history of a single parent and sibling. Similarly, the results show that individuals with a history of both the parents and siblings had an AOR of 2.1 and 3.3 for hypertension.

Moreover, findings further demonstrate that individuals with a medical history of father, mother, brother, and sister had significant 1.58, 1.64, 2.12, and 2.04 times higher odds of having hypertension, respectively, compared with those without a medical history.

**Figure 3:**
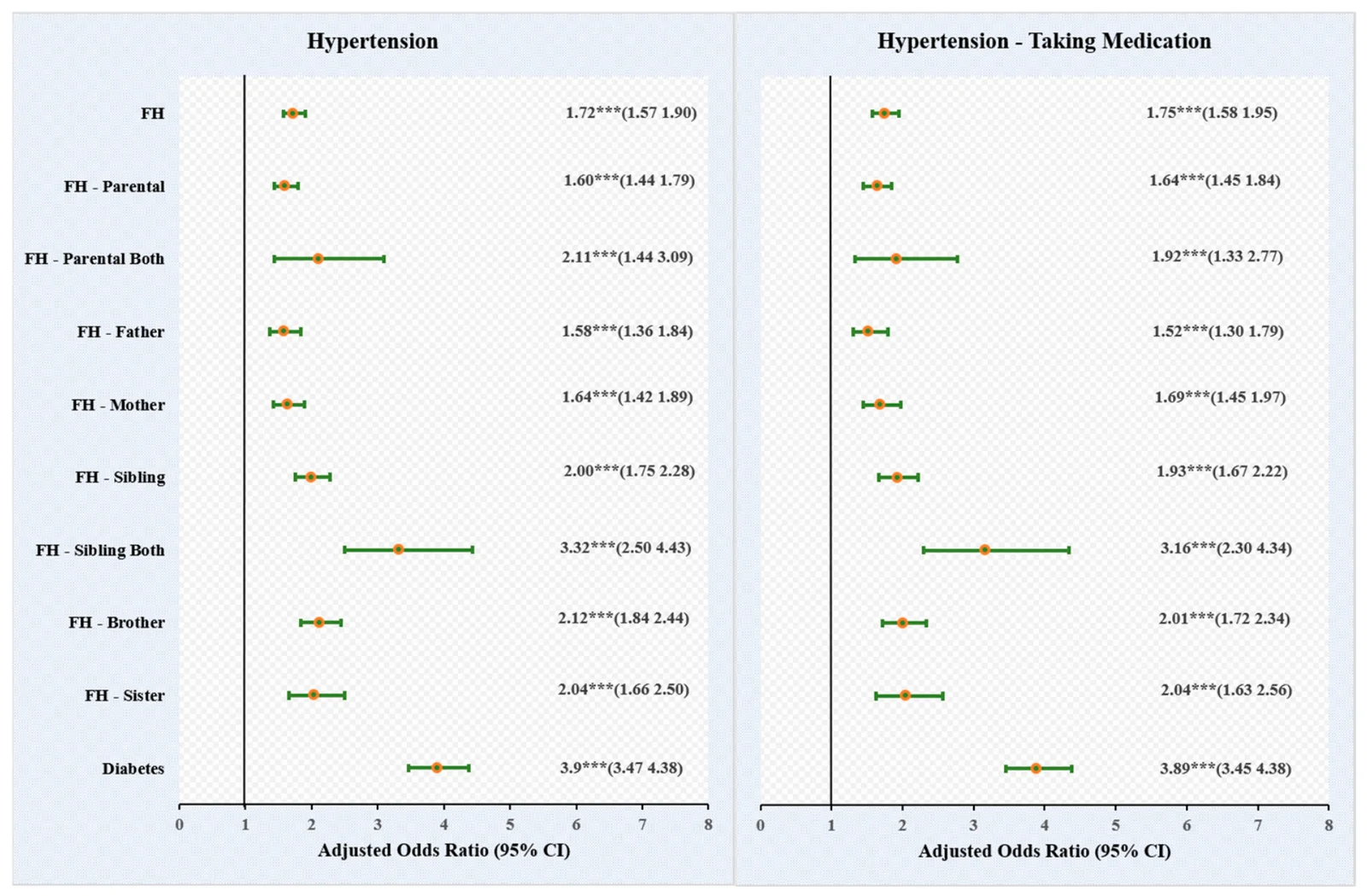
Multivariable logistic estimates for hypertension and hypertensive individuals taking medication among those aged 45 and above. *** *p* < 0.001; Ref, reference category; FH, family medical history of hypertension.

Figure 3 presents the unadjusted and adjusted logistic regression estimates for hypertension by family medical history of hypertension (overall, parental history, father, mother, sibling history, brother, and sister) and diabetes among individuals aged 45 years and above.

Table 5 presents the unadjusted and adjusted models of the additive interaction effect of family history of hypertension with diabetes on the diagnosis of hypertension. The current study provides estimates of the interaction effect on an additive scale using two different models of diabetes as an exposure variable (self-reported diabetes and individuals with diabetes taking treatments) for two different populations of hypertension (self-reported hypertension and hypertensive individuals receiving medication).

### 4.3. Interaction Effect of Family Medical History of Hypertension and Diabetes on the Diagnosis of Hypertension

#### 4.3.1. Self-Reported Diabetes and Family Medical History of Hypertension

The study findings show that the adjusted odds ratio (AOR) of the joint effect between a family medical history of hypertension and diabetes on the diagnosis of hypertension was 6.2 (95% CI: 5.08–7.58). In the adjusted model, the RERI, AP, and S values for hypertension were 1.39 (95% CI: 0.10–2.67), 22% (0.22; 95% CI: 0.06–0.40), and 1.36 (95% CI: 1.05–1.77) respectively, which indicates that there is a significant positive interaction effect between a family history of hypertension and diabetes on the diagnosis of hypertension.

#### 4.3.2. Self-Reported Diabetes (Taking Treatment) and Family Medical History of Hypertension

Moreover, the results support the interaction effect on all three measures based on individuals with diabetes who were taking medication. In the adjusted model, the RERI, AP, and S values for hypertension were 1.84 (95% CI: 0.41–3.27), 29% (0.29; 95% CI: 0.13–0.47), and 1.55 (95% CI: 1.15–2.09), respectively, which indicates that there is a significant positive interaction effect between a family history of hypertension and diabetes (taking medication) on the diagnosis of hypertension.

### 4.4. Interaction Effect Between Family Medical History of Hypertension and Diabetes on Hypertension (Taking Treatment for Hypertension)

The interaction effect of the family medical history of hypertension and diabetes is further supported by selecting the individuals with hypertension who were taking treatment.

#### 4.4.1. Self-Reported Diabetes and Family Medical History of Hypertension

Additionally, in this model, our findings support the interaction effect on all three measures based on individuals with diabetes who were taking medication. In the adjusted model, the RERI, AP, and S values for hypertension were 2.19 (95% CI: 0.86–3.52), 32% (0.32; 95% CI: 0.18–0.48), and 1.60 (95% CI: 1.25–2.06), respectively, which presents that there is a significant positive interaction effect between a family history of hypertension and diabetes (taking medication) on the diagnosis of hypertension.

#### 4.4.2. Self-Reported Diabetes (Taking Treatment) and Family Medical History of Hypertension

Furthermore, the study findings demonstrate that the adjusted odds ratio (AOR) of the joint effect between family medical history of hypertension and diabetes on the diagnosis of hypertension was 8.34 (95% CI: 6.73–10.33). In the adjusted model, the RERI was 3.09 (95% CI: 1.28–4.89), which indicates that there is a significant positive interaction effect between a family history and diabetes on the diagnosis of hypertension. The AP value was 37% (0.37; 95% CI: 0.22–0.52), which suggests that a significant proportion of individuals with hypertension in the population can be attributed to the interaction between a family medical history of hypertension and diabetes. The S value was 1.73 (95% CI: 1.31–2.27), further supporting a significant synergistic effect.

**Figure 4:**
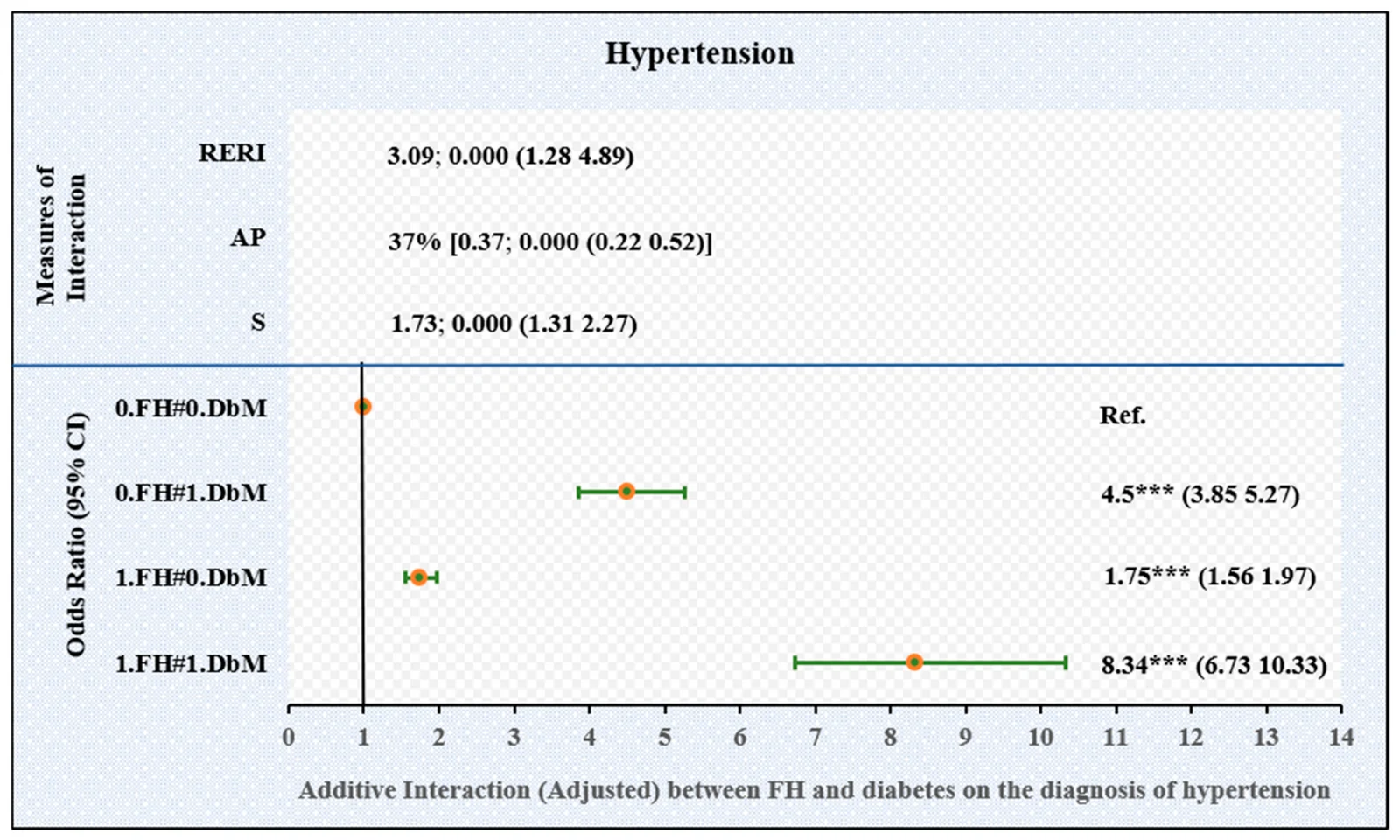
Additive interaction effect between family medical history of hypertension and diabetes (taking medication) on the diagnosis of hypertension (taking medication) among individuals aged 45 and above in India. *** *p* < 0.001; Ref, reference category; FH, family medical history of hypertension; DbM, individuals with diabetes who were on medication. Interaction exists if RERI ! = 0 or AP ! = 0 or S ! = 1. Adjusted for age, sex, education, working, marital status, residence, MPCE, religion, caste, region, physical inactivity, smoking, chewing tobacco, alcohol consumption, body mass index (BMI), ADL, IADL, high cholesterol, arthritis, and stroke. RERI, relative excess risk due to interaction; AP, attributable proportion; S, synergy index.

Figure 4 presents the additive interaction effect between a family medical history of hypertension and diabetes (taking medication) on the diagnosis of hypertension (taking medication) among individuals aged 45 and above in India.

## 5. Discussion

The study aimed to examine the additive interaction effect between a family medical history of hypertension and diabetes on the diagnosis of hypertension The current study demonstrates that, in the additive model, interaction effects between a family medical history of hypertension and diabetes on the risk of hypertension were significantly positive, as observed based on all three measures (RERI, AP and S), even after adjustment with potential confounding factors, supported by different models of sample selection. This suggests that the combined effect of a family medical history of hypertension and diabetes on the risk of developing hypertension is greater than the sum of their individual effects.

The study finding indicates that the prevalence of hypertension was more than 1.5 times higher among individuals with a family medical history of hypertension (39.4% vs. 23.8%) compared with those without a family medical history. Concordantly, previous studies reported that the prevalence of hypertension was notably higher among individuals with a family history of hypertension [10,34]. Additionally, a recent study found that 30.97% of hypertensive patients reported having a family history of hypertension [2]. Moreover, the results show that individuals with a family history of hypertension had an AOR of 1.7 for hypertension compared to individuals without a family history. Consistently, existing literature suggested that a family history of hypertension is significantly associated with an increased risk of developing hypertension [2,10,34,35]. Additionally, a prior study showed that the odds ratio for incident hypertension was 1.39 for individuals with any family history of hypertension compared to those without [36].

The study findings indicate that respondents with a parental and sibling medical history of hypertension had an AOR of 1.6 and 2.0 for hypertension, respectively, compared with those without a parental and sibling history. Concordantly, previous studies revealed that among adults, having a family history of hypertension in parents and siblings was significantly associated with an increased risk of hypertension [2,10]. A prior study showed that individuals with a family history of hypertension in both parents had an odds ratio of 3.05 for developing hypertension compared to those without a family history [36]. Consistently, our results demonstrate that individuals with a medical history of both parents and siblings had AORs of 2.1 and 3.3 for hypertension, respectively.

Importantly, the current study shows that both siblings with a family history of hypertension had higher odds of having hypertension, followed by both parents, any siblings, and any parents. Moreover, our findings present that individuals with a father, mother, brother, or sister medical history of hypertension had an odds ratio of 1.58, 1.64, 2.12, and 2.04 for hypertension, respectively, compared with those without family history hypertension after adjustment with the potential confounding factors. The presence of hypertension across multiple generations heightens the probability of hypertension onset in their offspring [36].

Further, study findings reveal that the prevalence of hypertension is higher among female respondents with a family medical history of hypertension than male respondents, including a parental, sibling, father, mother, brother, and sister medical history of hypertension. Additionally, hypertension was highly prevalent among female respondents with the coexistence of a family history and diabetes compared with male respondents. A previous study reported the differences in blood pressure trajectories between sexes [37]. Notably, females have shown a rapid progression of blood pressure measures over their life course, potentially indicating a sex-specific genetic predisposition to hypertension [37,38]. Interestingly, a study further demonstrated that these sex-based differences in blood pressure trajectories remained significant even after adjusting for multiple cardiovascular disease risk factors [37].

The current study further shows that the prevalence of hypertension was 2.8-fold higher among individuals with self-reported diabetes compared with those without diabetes. A prior study showed that hypertension was a prevalent complication of diabetes, with an approximate 20–30% incidence among patients with diabetes [39].

Furthermore, findings demonstrate that the AOR of the joint effect between a family medical history of hypertension and diabetes on the diagnosis of hypertension was 8.34 (95% CI: 6.73–10.33). Consistently, a prior study demonstrated that the odds ratio due to an interaction of the family history of hypertension with diabetes was 16.53 (95% CI 10.070–21.157) [24]. In the adjusted model, the RERI was 3.09 (95% CI: 1.28–4.89), which indicates that there is a significant positive interaction effect between a family history and diabetes on the diagnosis of hypertension. The AP value was 37% (0.37; 95% CI: 0.22–0.52), which suggests that a significant proportion of individuals with hypertension in the population can be attributed to the interaction between a family medical history of hypertension and diabetes. The S value was 1.73 (95% CI: 1.31–2.27), further supporting a significant synergistic effect.

In the additive model, the current study reveals that a family medical history of hypertension has a significantly positive synergistic interaction effect with diabetes on the risk of hypertension.

### Implication for Policy, Practice, and Future Research

Individuals with a high genetic risk of hypertension can be offset by adopting early favorable lifestyle modifications [40]. A positive family medical history of hypertension could be employed as a tool to improve risk counselling, especially when encouraging people to make lifestyle changes. Interestingly, newer glucose-lowering medications have been shown to be associated with weight loss, blood pressure reductions, and, most importantly, a reduced risk of major adverse cardiovascular events [41,42,43]. In addition, studies have shown that inhibitors of sodium-glucose cotransporter 2 (SGLT2 inhibitors), a class of drugs used to treat type 2 diabetes, can help to lower blood pressure and reduce the risk of cardiovascular events [43,44].

In a recent meta-analysis of 15 randomized controlled trials (RCTs), app-based behavioral self-monitoring interventions were found to yield a small but statistically significant reduction in systolic blood pressure among patients diagnosed with hypertension. Additionally, these interventions were associated with improved medication adherence behavior when compared to usual care or minimal intervention [45]. Moreover, a meta-analysis of 16 cohort studies revealed a linear dose–response relationship between adherence to blood pressure medication and CVD events, with each 20% increase in adherence linked to a 13% reduction in the risk of CVD events [46].

Further, in a meta-analysis of 133 studies, blood pressure reduction attained through sodium reduction exhibited a dose–response relationship, and reduction was more pronounced among older populations and individuals with elevated blood pressure levels [47]. Utilizing smartphone apps for the self-monitoring of hypertension-related behaviors, along with personalized guidance, results in a modest but significant clinical effect in the reduction of blood pressure [45].

The findings underscore the need for integrated screening and prevention strategies that simultaneously address multiple risk factors such as a family history of hypertension and diabetes. Health policies should prioritize comprehensive risk assessments in clinical settings to identify individuals at a higher risk due to the combined effects of these factors. Tailored intervention programs that account for the synergistic effects of these risk factors could enhance the effectiveness of hypertension prevention and treatment strategies. Additionally, investigating the potential benefits of combined lifestyle and pharmacological interventions in individuals with both risk factors could provide valuable insights into effective hypertension management.

## 6. Limitations and Strengths

The present study utilizes a cross-sectional survey design based on the first wave of the LASI data, which precludes establishing causal relationships. Additionally, the reliance on self-reported information may introduce reporting bias. However, it is notable that over seventy percent of the respondents reported receiving medication for hypertension, thereby reducing recall bias. These limitations should be considered when interpreting the study’s findings. Despite these constraints, the study has significant strengths. In LASI, respondents who reported being diagnosed with a disease by a medical professional were asked additional questions regarding the diagnosing physician, the date of diagnosis, and current medication usage. The study estimated the interaction effect on an additive scale for two models of diabetes (self-reported diabetes and individuals receiving treatment for diabetes) across two different samples (self-reported hypertension and individuals receiving treatment for hypertension). Moreover, this is the first large-scale population-based study to investigate the interaction effect of a family medical history of hypertension and diabetes on hypertension within the LMIC context, particularly in India. Additional research using robust study designs, such as prospective cohort studies or randomized controlled trials, is needed to further explore and validate the observed additive interactions. Further studies in diverse populations, including populations from high-income countries and other geographic regions, are needed to determine whether these observed associations are consistent across different genetic and environmental settings.

## 7. Conclusions

The current study reveals that in the additive model, a family medical history of hypertension has a significantly positive synergistic interaction effect with diabetes on the risk of hypertension, which indicates that the combined effect of a family medical history of hypertension and diabetes is larger than the sum of individuals effects on the risk of hypertension. A family medical history of hypertension can be utilized as a valuable tool to identify vulnerable subpopulations at a high risk of undiagnosed hypertension, especially among individuals with diabetes, which could help to develop more targeted and effective approaches to early diagnosis, personalized treatment, lifestyle modification, and treatment adherence leading to improved health outcomes.

## Figures and Tables

**Table 1:** Sample characteristics and percentage distribution of hypertension and its treatment by background characteristics among individuals aged 45 years and above, Longitudinal Ageing Study in India (LASI, 2017–18).

	Sample		Hypertension		Taking Treatment	
Background Characteristics	N	w col %	N	w Row % (95% CI)	N	w Row %
Sociodemographic Variables						
**Age groups**						
45–54	21,789	35.08	4602	19.6 (18.6, 20.6)	2859	60.27
55–64	18,229	30.45	5306	27.2 (25.9, 28.4)	3900	72.83
65–74	13,019	24.04	4726	34.2 (32.0, 36.4)	3673	77.86
75+	5630	10.43	2118	34.9 (32.5, 37.2)	1655	77.81
**Gender**						
Male	27,170	45.91	6605	22.6 (21.6, 23.6)	4699	70.12
Female	31,497	54.09	10,147	30.7 (29.5, 32.0)	7388	72.90
**Education level**						
No education	27,518	50.46	7077	23.9 (23.2, 24.7)	4781	66.79
Primary	14,661	23.43	4389	28.4 (27.1, 29.8)	3237	73.20
Secondary	10,863	16.28	3360	31.9 (28.8, 35.1)	2561	78.12
Higher	5625	9.83	1926	31.2 (27.6, 35.0)	1508	78.10
**Working status**						
Never worked	16,041	25.98	5845	35.5 (33.2, 37.7)	4461	75.97
Currently working	27,353	47.28	5413	18.6 (17.7, 19.5)	3451	63.50
Currently not working	15,273	26.73	5494	33.6 (32.4, 34.8)	4175	75.75
**Marital status**						
Currently married	43,971	73.94	11,634	24.7 (24.0, 25.5)	8246	69.80
Widowed	12,825	23.24	4631	34.9 (32.7, 37.2)	3503	76.94
D/S/D/Others	1871	2.81	487	21.3 (16.9,26.4)	338	64.83
**Place of residence**						
Rural	38,395	70.09	9312	22.7 (22.1, 23.3)	6104	65.50
Urban	20,272	29.91	7440	37.1 (34.9, 39.4)	5983	80.90
**Caste**						
Scheduled caste	9878	19.40	2577	23.9 (22.6,25.3)	1718	65.13
Scheduled tribe	10,264	8.64	2079	15.4 (14.2, 16.8)	1318	60.98
OBC	22,156	45.49	6322	27.0 (25.5, 28.6)	4632	73.24
Others	16,369	26.46	5774	32.9 (31.8, 34.1)	4419	75.07
**MPCE quintile**						
Poorest	11,550	21.00	2493	20.6 (19.4, 21.9)	1600	62.46
Poorer	11,858	21.28	3010	24.5 (23.2, 25.9)	2077	68.50
Middle	11,864	20.47	3389	26.2 (24.8, 27.7)	2446	71.30
Richer	11,843	19.69	3782	30.8 (28.6, 33.1)	2808	75.19
Richest	11,552	17.56	4078	34.2 (31.6, 36.9)	3156	78.56
**Region**						
North	10,812	12.70	3737	33.7 (32.5, 34.9)	2536	65.62
Central	8062	21.07	1545	19.2 (18.1, 20.4)	939	60.39
East	10,614	23.90	2696	25.4 (24.2, 26.6)	1741	63.56
Northeast	7580	3.38	1837	28.8 (27.2, 30.5)	1092	63.75
West	7706	15.93	2259	28.3 (26.7, 29.9)	1851	79.92
South	13,893	23.01	4678	30.9 (28.1, 33.8)	3928	85.14
**Religion**						
Hindu	43,060	82.50	11,737	25.9 (25.0, 26.9)	8399	71.10
Muslim	6948	11.04	2424	32.5 (30.3, 34.7)	1866	73.91
Christian	5876	2.95	1539	25.7 (21.3, 30.6)	1059	79.04
Others	2783	3.51	1052	35.6 (32.9, 38.4)	763	73.98
**Lifestyle factors**						
**Smoking tobacco**						
Never	47,882	82.72	14,279	28.2 (27.3, 29.2)	10,496	72.91
Former	2666	3.77	838	30.2 (26.9, 33.6)	610	71.80
Current	8119	13.50	1635	18.4 (17.2, 19.7)	981	61.79
**Chewing tobacco**						
Never	45,797	76.43	13,727	28.5 (27.5, 29.5)	10,109	73.31
Former	1436	2.38	438	30 (25.7, 34.6)	305	65.70
Current	11,434	21.19	2587	21.1 (19.9, 22.4)	1673	65.60
**Alcohol use**						
No	48,101	84.81	14,410	28.2 (27.2, 29.1)	10,577	72.80
Yes	10,566	15.19	2342	20.4 (19.2, 21.6)	1510	64.36
**Physical inactivity**						
Active	37,894	65.77	9971	25.0 (23.9, 26.1)	6979	69.73
Inactive	20,773	34.23	6781	30.9 (29.9, 31.9)	5108	75.10
**BMI categories**						
Normal	30,663	51.59	7750	24.2 (23.4, 25.0)	5321	68.15
Underweight	10,857	21.34	1776	15.3 (14.3, 16.3)	987	53.75
Overweight/obese	17,147	27.08	7226	41.5 (39.3, 43.8)	5779	81.17
**Morbidities**						
**Difficulty in ADL**						
No	50,732	84.48	13,651	25.5 (24.6, 26.4)	9664	70.60
Yes	7935	15.52	3101	35.1 (33.3, 36.9)	2423	76.73
**Difficulty in IADL**						
No	39,647	63.49	10,332	24.3 (23.4, 25.1)	7316	69.98
Yes	19,020	36.51	6420	31.7 (30.1, 33.4)	4771	74.30
**High cholesterol**						
No	56,591	97.75	15,308	26.1 (25.3, 26.9)	10,842	71.18
Yes	2076	2.25	1444	66.3 (60.9, 71.3)	1245	83.06
**Arthritis**						
No	53,888	90.64	14,785	25.6 (24.9, 26.3)	10,554	70.72
Yes	4779	9.36	1967	40.8 (35.9, 45.7)	1533	78.59
**Stroke**						
No	57,744	98.33	16,155	26.4 (25.6, 27.3)	11,590	71.34
Yes	923	1.67	597	61.2 (56.7, 65.6)	497	84.38
**Diabetes**						
No	51,184	88.09	12,194	22.2 (21.6, 22.9)	8209	66.70
Yes	7483	11.91	4558	62.2 (59.3, 65.0)	3878	85.41
Treatment for Db *						
No	1308	17.49	780	59.6 (55.5, 63.6)	414	47.70
Yes	6175	82.51	3778	62.8 (59.3, 66.1)	3464	93.01
**Treatment for Db**						
No	52,492	90.17	12,974	23.1 (22.4, 23.8)	8623	65.56
Yes	6175	9.83	3778	62.8 (59.3, 66.1)	3464	93.01
**Family Medical History**						
**Family history of hypertension**						
No	45,906	79.50	11,341	23.8 (23.1, 24.5)	7920	69.50
Yes	12,761	20.50	5411	39.4 (36.6, 42.2)	4167	77.29
**Total**	**58,667**	**100.00**	**16,752**	**27.0 (26.2, 27.8)**	**12,087**	**71.83**

Notes: N: Sample; w %: weighted percentages to account for survey design and to provide national population estimates; ADL: activities of daily living; IADL: instrumental activities of daily living; MPCE: monthly per capita consumption expenditure; Db, diabetes; *, treatment for diabetes among individuals with self-reported diabetes.

**Table 2:** Frequencies and percentage prevalence of hypertension and its treatment by family medical history of hypertension.

	Sample		Hypertension			Taking Medication		
Family Medical History of Hypertension	N	w col %	N	w Row % (95% CI)	*p*-Value	N	w Row %	*p*-Value
**FH-Parental**					0.000			0.000
No	49,058	84.38	12,685	24.9 (24.2, 25.8)		8955	70.45	
Yes	9609	15.62	4067	38.1 (35.0, 41.2)		3132	76.74	
**FH-Parental both**					0.000			0.000
No	57,018	97.61	15,859	26.5 (25.7, 27.3)		11,392	71.67	
Yes	1649	2.39	893	47.2 (37.8, 56.7)		695	75.59	
**FH-Father**					0.000			0.000
No	53,469	91.83	14,430	25.9 (25.1, 26.8)		10,308	71.46	
Yes	5198	8.17	2322	38.9 (35.4, 42.6)		1779	74.63	
**FH-Mother**					0.000			0.000
No	52,607	90.17	14,114	25.6 (24.9, 26.4)		10,039	70.77	
Yes	6060	9.83	2638	39.5 (35.2, 44.0)		2048	78.14	
**FH-Sibling**					0.000			0.000
No	53,922	92.63	14,543	25.3 (24.6, 26.0)		10,369	70.61	
Yes	4745	7.37	2209	48.5 (43.4, 53.7)		1718	79.86	
**FH-Sibling both**					0.000			0.000
No	57,959	98.84	16,322	26.5 (25.7, 27.3)		11,723	71.33	
Yes	708	1.16	430	69.7 (56.0, 80.6)		364	88.07	
**FH-Brother**					0.000			0.000
No	55,252	94.49	15,118	25.6 (24.9, 26.3)		10,820	70.82	
Yes	3415	5.51	1634	51.5 (45.2, 57.6)		1267	80.45	
**FH-Sister**					0.000			0.000
No	56,881	96.98	15,747	26.2 (25.5, 27.0)		11,272	71.15	
Yes	1773	3.02	1005	51.4 (43.0, 59.6)		815	83.09	
**Total**	**58,667**	**100.00**	**16,752**	**27.0 (26.2, 27.8)**		**12,087**	**71.83**	

Notes: N: Sample; w %: weighted percentages to account for survey design and to provide national population estimates; FH, family medical history of hypertension.

**Table 3:** The prevalence of hypertension by key predictor variables among individuals aged 45 and above.

	Hypertension			Hypertension–Currently on Medication		
Variables	N	Percentage (95% CI)	*p*-Value	N	Percentage (95% CI)	*p*-Value
**FH and Diabetes**			0.000			0.000
No	15,063	25.3 (24.6, 26.0)		10,595	17.6 (17.0, 18.3)	
Yes	1689	70.7 (64.6, 76.1)		1492	64.3 (57.3, 70.7)	
**FH#Diabetes**						0.000
0.FH#0.diabetes	8472	19.8 (19.2, 20.5)	0.000	5534	12.9 (12.4, 13.5)	
0.FH#1.diabetes	2869	58.3 (55.6, 61.0)		2386	48.0 (45.1, 50.8)	
1.FH#0.diabetes	3722	32.4 (30.3, 34.7)		2675	22.9 (21.1, 24.8)	
1.FH#1.diabetes	1689	70.7 (64.6, 76.1)		1492	64.3 (57.3, 70.7)	
**FH and Diabetes_Medication**			0.000			0.000
No	15,305	25.5 (24.8, 26.2)		10,730	17.7 (17.1, 18.3)	
Yes	1447	72.7 (66.0, 78.5)		1357	69.7 (62.6, 76.1)	
**FH#Diabetes_Medication**			0.000			0.000
0.FH#0.diabetes_M	9010	20.7 (20.0, 21.4)		5813	13.2 (12.7, 13.8)	
0.FH#1.diabetes_M	2331	58.0 (54.8, 61.1)		2107	52.8 (49.6, 56.1)	
1.FH#0.diabetes_M	3964	33.3 (31.1, 35.5)		2810	23.2 (21.5, 25.0)	
1.FH#1.diabetes_M	1447	72.7 (66.0, 78.5)		1357	69.7 (62.6, 76.1)	
**Total**	**16,752**	**27.0 (26.2, 27.8)**		**12,087**	**19.4 (18.6, 20.2)**	

FH, family medical history of hypertension; diabetes_M, individuals with diabetes who were taking medication. 0.FH#0.diabetes, absence of family medical history of hypertension # absence of diabetes; 0.FH#1.diabetes, absence of family medical history of hypertension # presence of diabetes; 1.FH#0.diabetes, presence of family medical history of hypertension # absence of diabetes; 1.FH#1.diabetes, presence of family medical history of hypertension # presence of diabetes.

**Table 4:** Multivariable logistic regression estimates for hypertension and its treatment by key predictor variables among individuals aged 45 and above in India.

	Hypertension		Hypertension–Taking Treatment	
Background Characteristics	UOR (95% CI)	AOR (95% CI)	UOR (95% CI)	AOR (95% CI)
**Family Medical History of Hypertension**				
No	Ref.	Ref.	Ref.	Ref.
Yes	2.08 *** (1.84 2.36)	1.72 *** (1.57 1.90)	2.21 *** (1.92 2.55)	1.75 *** (1.58 1.95)
**FH-Parental**				
No	Ref.	Ref.	Ref.	Ref.
Yes	1.85 *** (1.61 2.12)	1.60 *** (1.44 1.79)	1.94 *** (1.66 2.26)	1.64 *** (1.45 1.84)
**FH-Parental-both**				
No	Ref.	Ref.	Ref.	Ref.
Yes	2.48 *** (1.68 3.64)	2.11 *** (1.44 3.09)	2.36 *** (1.69 3.30)	1.92 *** (1.33 2.77)
**FH-Father**				
No	Ref.	Ref.	Ref.	Ref.
Yes	1.82 *** (1.55 2.14)	1.58 *** (1.36 1.84)	1.80 *** (1.55 2.10)	1.52 *** (1.30 1.79)
**FH-Mother**				
No	Ref.	Ref.	Ref.	Ref.
Yes	1.90 *** (1.57 2.29)	1.64 *** (1.42 1.89)	2.02 *** (1.64 2.49)	1.69 *** (1.45 1.97)
**FH-Sibling**				
No	Ref.	Ref.	Ref.	Ref.
Yes	2.79 *** (2.26 3.43)	2.00 *** (1.75 2.28)	2.91 *** (2.27 3.75)	1.93 *** (1.67 2.22)
**FH-Sibling-both**				
No	Ref.	Ref.	Ref.	Ref.
Yes	6.40 *** (3.53 11.57)	3.32 *** (2.50 4.43)	6.84 *** (3.54 13.21)	3.16 *** (2.30 4.34)
**FH-Brother**				
No	Ref.	Ref.	Ref.	Ref.
Yes	3.09 *** (2.40 3.97)	2.12 *** (1.84 2.44)	3.20 *** (2.36 4.33)	2.01 *** (1.72 2.34)
**FH-Sister**				
No	Ref.	Ref.	Ref.	Ref.
Yes	2.97 *** (2.12 4.16)	2.04 *** (1.66 2.50)	3.25 *** (2.20 4.79)	2.04 *** (1.63 2.56)
**Diabetes**				
No	Ref.	Ref.	Ref.	Ref.
Yes	5.76 *** (5.06 6.55)	3.90 *** (3.47 4.38)	6.51 *** (5.66 7.48)	3.89 *** (3.45 4.38)

*** *p* < 0.001; Ref, reference category; FH, family medical history; UOR, unadjusted odds ratio; AOR, adjusted odds ratio, adjusted for age, sex, education, marital status, working status, residence, caste, MPCE, region, religion, smoking, chewing tobacco, alcohol, use, physical activity, body mass index, ADL, IADL, and high cholesterol, arthritis, and stroke.

**Table 5:** Additive interaction effect between family medical history of hypertension and diabetes baa on the diagnosis of hypertension among individuals aged 45 and above.

	Unadjusted (95% CI)	Adjusted (95% CI)
**(1) Self-reported hypertension**		
***(A) Self-reported Diabetes***		
**Additive Interaction between family history and diabetes**		
0.FH#0.diabetes	Ref.	Ref.
0.FH#1.diabetes	5.66 *** (5.02 6.37)	4.05 *** (3.55 4.62)
1.FH#0.diabetes	1.94 *** (1.74 2.16)	1.77 *** (1.59 1.97)
1.FH#1.diabetes	9.74 *** (7.34 12.91)	6.20 *** (5.08 7.58)
**Measures of interaction on the additive scale (** ***p*** **-value, 95% CI)**		
**RERI**	**3.14**; 0.028 (0.34 5.94)	**1.39**; 0.034 (0.10 2.67)
**AP ^$^**	**32.00%** [0.32; 0.002 (0.12 0.52)]	**22.00%** [0.22; 0.009 (0.06 0.40)]
**SI**	**1.56**; 0.009 (1.12 2.18)	**1.36**; 0.021 (1.05 1.77)
***(B) Self-reported Diabetes—currently on medication***		
**Additive interaction between family history and diabetes with medications**		
0.FH#0.diabetes_M	Ref.	Ref.
0.FH#1.diabetes_M	5.29 *** (4.62 6.07)	3.60 *** (3.09 4.17)
1.FH#0.diabetes_M	1.91 *** (1.72 2.13)	1.74 *** (1.57 1.93)
1.FH#1.diabetes_M	10.21 *** (7.44 14.02)	6.17 *** (4.93 7.72)
**Measures of interaction on the additive scale (** ***p*** **-value, 95% CI)**		
**RERI**	**4.01**; 0.017 (0.72 7.23)	**1.84**; 0.012 (0.41 3.27)
**AP ^$^**	**39%** [0.39; 0.002 (0.19 0.60)]	**29%** [0.29; 0.000 (0.13 0.47)]
**SI**	**1.77**; 0.028 (1.22 2.57)	**1.55**; 0.004 (1.15 2.09)
**(2) Self-reported hypertension—currently on medication**		
***(C) Self-reported Diabetes***		
**Additive interaction between family history and diabetes**		
0.FH#0.diabetes	Ref.	Ref.
0.FH#1.diabetes	6.21 *** (5.48 7.05)	3.88 *** (3.37 4.47)
1.FH#0.diabetes	2.00 *** (1.78 2.24)	1.75 *** (1.55 1.98)
1.FH#1.diabetes	12.12 *** (8.98 16.36)	6.83 *** (5.63 8.28)
**Measures of interaction on the additive scale (** ***p*** **-value, 95% CI)**		
**RERI**	**4.91**; 0.009 (1.25 8.57)	**2.19**; 0.001 (0.86 3.52)
**AP ^$^**	**40%** [0.40; 0.000 (0.22 0.59)]	**32%** [0.32; 0.000 (0.18 0.46)]
**SI**	**1.79**; 0.000 (1.27 2.52)	**1.60**; 0.000 (1.25 2.06)
***(D) Self-reported Diabetes—currently on medication***		
**Additive interaction between family history and diabetes with medications**		
0.FH#0.diabetes_M	Ref.	Ref.
0.FH#1.diabetes_M	7.35 *** (6.39 8.46)	4.50 *** (3.85 5.27)
1.FH#0.diabetes_M	1.98 *** (1.78 2.22)	1.75 *** (1.56 1.97)
1.FH#1.diabetes_M	15.13 *** (10.93 20.94)	8.34 *** (6.73 10.33)
**Measures of interaction on the additive scale (** ***p*** **-value, 95% CI)**		
**RERI**	**6.79**; 0.007 (1.82 11.75)	**3.09**; 0.000 (1.28 4.89)
**AP ^$^**	**45%** [0.45; 0.000 (0.26 0.64)]	**37%** [0.37; 0.000 (0.22 0.52)]
**SI**	**1.92**; 0.000 (1.33 2.78)	**1.73**; 0.000 (1.31 2.27)

*** *p* < 0.001; Ref, reference category; FH, family medical history of hypertension; HT, hypertension; diabetes_M, individuals with diabetes who were on medication. Interaction exists if RERI ! = 0 or AP ! = 0 or S ! = 1; Model 1: unadjusted model; Model 2: adjusted for age, sex, education, working, marital status, residence, MPCE, religion, caste, region, physical inactivity, smoking, chewing tobacco, alcohol consumption, body mass index (BMI), ADL, IADL, high cholesterol, arthritis, and stroke; RERI, relative excess risk due to interaction; AP, attributable proportion; AP ^$^, the attributable proportion, has been presented in the result as a percentage after multiplied by 100; S, synergy index. 0.FH#0.diabetes: absence of family medical history of hypertension # absence of diabetes; 0.FH#1.diabetes: absence of family medical history of hypertension # presence of diabetes; 1.FH#0.diabetes: presence of family medical history of hypertension # absence of diabetes; 1.FH#1.diabetes: presence of family medical history of hypertension # presence of diabetes.

## Data Availability

The study uses secondary data, which are available for academic research purposes, and the request can be made or accessible through https://www.iipsindia.ac.in/content/LASI-data (accessed on 14 October 2026).

## Abbreviations

**RERI**	Relative excess risk due to interaction
**AP**	Attribution proportion
**S**	Synergy index
**FH**	Family medical history

## References

[1] Warren H.R., Evangelou E., Cabrera C.P., Gao H., Ren M., Mifsud B., Ntalla I., Surendran P., Liu C., Cook J.P. (2017). Genome-Wide Association Analysis Identifies Novel Blood Pressure Loci and Offers Biological Insights into Cardiovascular Risk. Nat. Genet..

[2] Kc K., Katwal S., Yadav G.K., Adhikari A., Thapa R.K., Jha S.K., Sharma A., Rijal T., Giri S., Khadka S. (2023). Family History of Hypertension and Its Relation to Other Variables in Hypertensive Patients: A Cross-Sectional Study from a Tertiary Care Hospital. Int. J. Surg. Glob. Health.

[3] Shih P.B., O’Connor D.T. (2008). Hereditary Determinants of Human Hypertension. Hypertension.

[4] Snieder H., Hayward C.S., Perks U., Kelly R.P., Kelly P.J., Spector T.D. (2000). Heritability of Central Systolic Pressure Augmentation. Hypertension.

[5] Hopkins P.N., Hunt S.C. (2003). Genetics of Hypertension. Genet. Med..

[6] Seidel E., Scholl U.I. (2017). Genetic Mechanisms of Human Hypertension and Their Implications for Blood Pressure Physiology. Physiol. Genom..

[7] Tozawa M., Oshiro S., Iseki C., Sesoko S., Higashiuesato Y., Tana T., Ikemiya Y., Iseki K., Fukiyama K. (2001). Family History of Hypertension and Blood Pressure in a Screened Cohort. Hypertens. Res..

[8] Fuentes R., Notkola I.L., Shemeikka S., Tuomilehto J., Nissinen A. (2000). Familial Aggregation of Blood Pressure: A Population-Based Family Study in Eastern Finland. J. Hum. Hypertens..

[9] Goldstein I.B., Shapiro D., Weiss R.E. (2008). How Family History and Risk Factors for Hypertension Relate to Ambulatory Blood Pressure in Healthy Adults. J. Hypertens..

[10] Ranasinghe P., Cooray D.N., Jayawardena R., Katulanda P. (2015). The Influence of Family History of Hypertension on Disease Prevalence and Associated Metabolic Risk Factors among Sri Lankan Adults. BMC Public Health.

[11] Jia P., Zhan N., Bat B.K.K., Feng Q., Tsoi K.K.F. (2022). The Genetic Architecture of Blood Pressure Variability: A Genome-Wide Association Study of 9370 Participants from UK Biobank. J. Clin. Hypertens..

[12] Gouveia M.H., Bentley A.R., Leonard H., Meeks K.A.C., Ekoru K., Chen G., Nalls M.A., Simonsick E.M., Tarazona-Santos E., Lima-Costa M.F. (2021). Trans-Ethnic Meta-Analysis Identifies New Loci Associated with Longitudinal Blood Pressure Traits. Sci. Rep..

[13] Parcha V., Pampana A., Shetty N.S., Irvin M.R., Natarajan P., Lin H.J., Guo X., Rich S.S., Rotter J.I., Li P. (2022). Association of a Multiancestry Genome-Wide Blood Pressure Polygenic Risk Score With Adverse Cardiovascular Events. Circ. Genom. Precis. Med..

[14] Tsimihodimos V., Gonzalez-Villalpando C., Meigs J.B., Ferrannini E. (2018). Hypertension and Diabetes Mellitus. Hypertension.

[15] Ali M.K., Bullard K.M., Saydah S., Imperatore G., Gregg E.W. (2018). Cardiovascular and Renal Burdens of Prediabetes in the USA: Analysis of Data from Serial Cross-Sectional Surveys, 1988–2014. Lancet Diabetes Endocrinol..

[16] Petrie J.R., Guzik T.J., Touyz R.M. (2018). Diabetes, Hypertension, and Cardiovascular Disease: Clinical Insights and Vascular Mechanisms. Can. J. Cardiol..

[17] Bundy J.D., Li C., Stuchlik P., Bu X., Kelly T.N., Mills K.T., He H., Chen J., Whelton P.K., He J. (2017). Systolic Blood Pressure Reduction and Risk of Cardiovascular Disease and Mortality: A Systematic Review and Network Meta-Analysis. JAMA Cardiol..

[18] Clark D., Colantonio L.D., Min Y.-I., Hall M.E., Zhao H., Mentz R.J., Shimbo D., Ogedegbe G., Howard G., Levitan E.B. (2019). Population-Attributable Risk for Cardiovascular Disease Associated With Hypertension in Black Adults. JAMA Cardiol..

[19] Navar A.M., Peterson E.D., Wojdyla D., Sanchez R.J., Sniderman A.D., D’Agostino R.B., Pencina M.J. (2016). Temporal Changes in the Association between Modifiable Risk Factors and Coronary Heart Disease Incidence. JAMA.

[20] Wan E.Y.F., Fung W.T., Schooling C.M., Au Yeung S.L., Kwok M.K., Yu E.Y.T., Wang Y., Chan E.W.Y., Wong I.C.K., Lam C.L.K. (2021). Blood Pressure and Risk of Cardiovascular Disease in UK Biobank. Hypertension.

[21] Levy D., Ehret G.B., Rice K., Verwoert G.C., Launer L.J., Dehghan A., Glazer N.L., Morrison A.C., Johnson A.D., Aspelund T. (2009). Genome-Wide Association Study of Blood Pressure and Hypertension. Nat. Genet..

[22] Solini A., Penno G., Bonora E., Fondelli C., Orsi E., Arosio M., Trevisan R., Vedovato M., Cignarelli M., Andreozzi F. (2012). Diverging Association of Reduced Glomerular Filtration Rate and Albuminuria with Coronary and Noncoronary Events in Patients with Type 2 Diabetes: The Renal Insufficiency and Cardiovascular Events (RIACE) Italian Multicenter Study. Diabetes Care.

[23] Ferrannini E., Cushman W.C. (2012). Diabetes and Hypertension: The Bad Companions. Lancet.

[24] Li A., Peng Q., Shao Y., Fang X., Zhang Y. (2021). The Interaction on Hypertension between Family History and Diabetes and Other Risk Factors. Sci. Rep..

[25] World Health Organization Nutrition for a Healthy Life—WHO Recommendations. https://www.who.int/europe/news-room/fact-sheets/item/nutrition---maintaining-a-healthy-lifestyle.

[26] Ahmed W., Muhammad T., Muneera K. (2023). Prevalence of Early and Late Onset of Chronic Diseases and Multimorbidity and Its Association with Physical, Mental and Functional Health among Older Indian Adults. BMC Geriatr..

[27] Ahmed W. (2024). Additive Interaction of Family Medical History of Diabetes with Hypertension on the Diagnosis of Diabetes among Older Adults in India: Longitudinal Ageing Study in India. BMC Public Health.

[28] Piercy K.L., Troiano R.P., Ballard R.M., Carlson S.A., Fulton J.E., Galuska D.A., George S.M., Olson R.D. (2018). The Physical Activity Guidelines for Americans. JAMA.

[29] World Health Organization WHO Guidelines on Physical Activity and Sedentary Behaviour. https://www.who.int/publications-detail-redirect/9789240015128.

[30] Knol M.J., VanderWeele T.J., Groenwold R.H.H., Klungel O.H., Rovers M.M., Grobbee D.E. (2011). Estimating Measures of Interaction on an Additive Scale for Preventive Exposures. Eur. J. Epidemiol..

[31] VanderWeele T.J., Knol M.J. (2014). A Tutorial on Interaction. Epidemiol. Methods.

[32] Rothman K.J., Greenland S., Lash T.L. (2008). Modern Epidemiology.

[33] King J.E., Osborne J. (2011). Binary Logistic Regression. Best Practices in Quantitative Methods.

[34] Liu M., He Y., Jiang B., Wang J., Wu L., Wang Y., Zhang D., Zeng J., Yao Y. (2015). Association Between Family History and Hypertension Among Chinese Elderly. Medicine.

[35] Bartwal J., Awasthi S., Rawat C.M.S., Singh R.K. (2014). Prevalence of Hypertension and Its Risk Factors among Individuals Attending Outpatient Department of Rural Health Training Centre, Haldwani. Indian J. Community Health.

[36] Igarashi R., Fujihara K., Heianza Y., Ishizawa M., Kodama S., Saito K., Hara S., Hanyu O., Honda R., Tsuji H. (2016). Impact of Individual Components and Their Combinations within a Family History of Hypertension on the Incidence of Hypertension: Toranomon Hospital Health Management Center Study 22. Medicine.

[37] Ji H., Kim A., Ebinger J.E., Niiranen T.J., Claggett B.L., Bairey Merz C.N., Cheng S. (2020). Sex Differences in Blood Pressure Trajectories Over the Life Course. JAMA Cardiol..

[38] Kauko A., Aittokallio J., Vaura F., Ji H., Ebinger J.E., Niiranen T., Cheng S. (2021). Sex Differences in Genetic Risk for Hypertension. Hypertension.

[39] Liu J., Zhao D., Liu J., Qi Y., Sun J., Wang W. (2013). Prevalence of Diabetes Mellitus in Outpatients with Essential Hypertension in China: A Cross-Sectional Study. BMJ Open.

[40] Martin S.S., Aday A.W., Almarzooq Z.I., Anderson C.A.M., Arora P., Avery C.L., Baker-Smith C.M., Barone Gibbs B., Beaton A.Z., Boehme A.K. (2024). 2024 Heart Disease and Stroke Statistics: A Report of US and Global Data from the American Heart Association. Circulation.

[41] Marso S.P., Daniels G.H., Brown-Frandsen K., Kristensen P., Mann J.F.E., Nauck M.A., Nissen S.E., Pocock S., Poulter N.R., Ravn L.S. (2016). Liraglutide and Cardiovascular Outcomes in Type 2 Diabetes. N. Engl. J. Med..

[42] Petrie J.R. (2013). The Cardiovascular Safety of Incretin-Based Therapies: A Review of the Evidence. Cardiovasc. Diabetol..

[43] Zinman B., Wanner C., Lachin J.M., Fitchett D., Bluhmki E., Hantel S., Mattheus M., Devins T., Johansen O.E., Woerle H.J. (2015). Empagliflozin, Cardiovascular Outcomes, and Mortality in Type 2 Diabetes. N. Engl. J. Med..

[44] Wiviott S.D., Raz I., Bonaca M.P., Mosenzon O., Kato E.T., Cahn A., Silverman M.G., Zelniker T.A., Kuder J.F., Murphy S.A. (2019). Dapagliflozin and Cardiovascular Outcomes in Type 2 Diabetes. N. Engl. J. Med..

[45] Kassavou A., Wang M., Mirzaei V., Shpendi S., Hasan R. (2022). The Association Between Smartphone App-Based Self-Monitoring of Hypertension-Related Behaviors and Reductions in High Blood Pressure: Systematic Review and Meta-Analysis. JMIR MHealth UHealth.

[46] Feng Y., Zhao Y., Yang X., Li Y., Han M., Qie R., Huang S., Wu X., Zhang Y., Wu Y. (2022). Adherence to Antihypertensive Medication and Cardiovascular Disease Events in Hypertensive Patients: A Dose–Response Meta-Analysis of 2,769,700 Participants in Cohort Study. QJM Int. J. Med..

[47] Huang L., Trieu K., Yoshimura S., Neal B., Woodward M., Campbell N.R.C., Li Q., Lackland D.T., Leung A.A., Anderson C.A.M. (2020). Effect of Dose and Duration of Reduction in Dietary Sodium on Blood Pressure Levels: Systematic Review and Meta-Analysis of Randomised Trials. BMJ.

